# p38 MAPK Is Activated but Does Not Play a Key Role during Apoptosis Induction by Saturated Fatty Acid in Human Pancreatic β-Cells

**DOI:** 10.3390/ijms17020159

**Published:** 2016-02-05

**Authors:** Jan Šrámek, Vlasta Němcová-Fürstová, Kamila Balušíková, Petr Daniel, Michael Jelínek, Roger F. James, Jan Kovář

**Affiliations:** 1 Division of Cell and Molecular Biology & Center for Research of Diabetes, Metabolism and Nutrition, Third Faculty of Medicine, Charles University in Prague, Ruská 87, Prague 110 00, Czech Republic; vlasta.furstova@tiscali.cz (V.N.-F.); kamila.balusikova@lf3.cuni.cz (K.B.); petr85daniel@gmail.com (P.D.); michael.j@email.cz (M.J.); 2 Department of Infection, Immunity and Inflammation, Maurice Shock Medical Sciences Building, University of Leicester, University Road, Leicester LE1 9HN, UK; rj1@leicester.ac.uk

**Keywords:** p38 MAPK, ERK, fatty acids, pancreatic β-cells, apoptosis, NES2Y

## Abstract

Saturated stearic acid (SA) induces apoptosis in the human pancreatic β-cells NES2Y. However, the molecular mechanisms involved are unclear. We showed that apoptosis-inducing concentrations of SA activate the p38 MAPK signaling pathway in these cells. Therefore, we tested the role of p38 MAPK signaling pathway activation in apoptosis induction by SA in NES2Y cells. Crosstalk between p38 MAPK pathway activation and accompanying ERK pathway inhibition after SA application was also tested. The inhibition of p38 MAPK expression by siRNA silencing resulted in a decrease in MAPKAPK-2 activation after SA application, but it had no significant effect on cell viability or the level of phosphorylated ERK pathway members. The inhibition of p38 MAPK activity by the specific inhibitor SB202190 resulted in inhibition of MAPKAPK-2 activation and noticeable activation of ERK pathway members after SA treatment but in no significant effect on cell viability. p38 MAPK overexpression by plasmid transfection produced an increase in MAPKAPK-2 activation after SA exposure but no significant influence on cell viability or ERK pathway activation. The activation of p38 MAPK by the specific activator anisomycin resulted in significant activation of MAPKAPK-2. Concerning the effect on cell viability, application of the activator led to apoptosis induction similar to application of SA (PARP cleavage and caspase-7, -8, and -9 activation) and in inhibition of ERK pathway members. We demonstrated that apoptosis-inducing concentrations of SA activate the p38 MAPK signaling pathway and that this activation could be involved in apoptosis induction by SA in the human pancreatic β-cells NES2Y. However, this involvement does not seem to play a key role. Crosstalk between p38 MAPK pathway activation and ERK pathway inhibition in NES2Y cells seems likely. Thus, the ERK pathway inhibition by p38 MAPK activation does not also seem to be essential for SA-induced apoptosis.

## 1. Introduction

Increased levels of fatty acids (FAs) in blood are considered to be one of the main factors responsible for pancreatic β-cell death in type 2 diabetes [1,2,3,4,5]. Our previous studies as well as others have shown that saturated FAs induce apoptosis in pancreatic β-cells [2,4,5,6,7,8]. Although the precise molecular mechanisms of apoptosis induction by FAs in β-cells remain unclear [9], there are some indications that the p38 MAPK (mitogen-activated protein kinase) signaling pathway could be involved [10,11].

p38 MAPK kinase has been shown to participate in the regulation of many cellular processes such as cell proliferation, differentiation, the inflammatory response, and apoptosis (reviewed in [12]). Generally, p38 MAPK is activated via the dual-specific MKK3/6 (mitogen-activated protein kinase kinase 3 and 6), kinase in response to various extracellular stimuli such as physical and chemical stress (reviewed in [12]). p38 MAPK regulates activation of MAPKAPK-2 (MAPK-activated protein kinase 2), which is responsible for nuclear export of activated p38 MAPK [13] and can also affect activation of certain proteins involved in apoptosis regulation such as NF-κB (nuclear factor κB) [14] or caspase-3 [15]. Concerning apoptosis, it has been demonstrated, in different cell types and under different experimental conditions, that p38 MAPK can mediate pro-apoptotic signaling [16,17]. The pro-apoptotic function of p38 MAPK has also been demonstrated in studies using pancreatic β-cells exposed to FAs. However, the possible role of p38 MAPK in FA-induced apoptosis in pancreatic β-cells remains unclear [10,18,19].

Several studies [16,20] have shown that in some types of cells, p38 MAPK can inhibit the c-Raf → MEK1/2 → ERK1/2 (extracellular signal-regulated kinases 1 and 2) pathway, *i.e.*, the ERK signaling pathway. The ERK pathway is mostly activated by growth factors. These factors, acting through receptor kinases and adaptor protein son of sevenless (SOS), activate Ras GTPase, which is responsible for c-Raf phosphorylation [21]. Like p38 MAPK, the ERK pathway can affect various proteins associated with apoptosis, e.g., Fox03a or several proteins of the Bcl-2 family [22]. It has been demonstrated that the ERK pathway can mediate both pro-apoptotic as well as anti-apoptotic signaling [23,24,25,26]. Regulation of ERK1/2 activation by saturated FAs in pancreatic β-cells has also been shown [27,28].

There are data supporting the idea that the p38 MAPK signaling pathway could be involved in apoptosis induction by FAs in pancreatic β-cells [10,18]. In the present study, we demonstrated that p38 MAPK is activated during apoptosis induction by stearic acid (SA) in the human pancreatic β-cells NES2Y. Therefore, we tested the role of p38 MAPK signaling pathway activation in apoptosis induction by SA, representing saturated FAs, in NES2Y cells. Crosstalk between p38 MAPK pathway activation and ERK pathway inhibition, after SA application, was also tested. We demonstrated that the activation of the p38 MAPK pathway could be somehow involved in apoptosis induction by SA in the human pancreatic β-cells NES2Y. However, this involvement does not seem to play a key role. Crosstalk between p38 MAPK pathway activation and ERK pathway inhibition in NES2Y cells seems likely. Thus, the ERK pathway inhibition by p38 MAPK activation does not also seem to be essential for SA-induced apoptosis.

## 2. Results

### 2.1. Effect of Stearic Acid on Cell Death Induction and Activation of Members of the p38 MAPK and ERK Signaling Pathways

**Figure 1:**
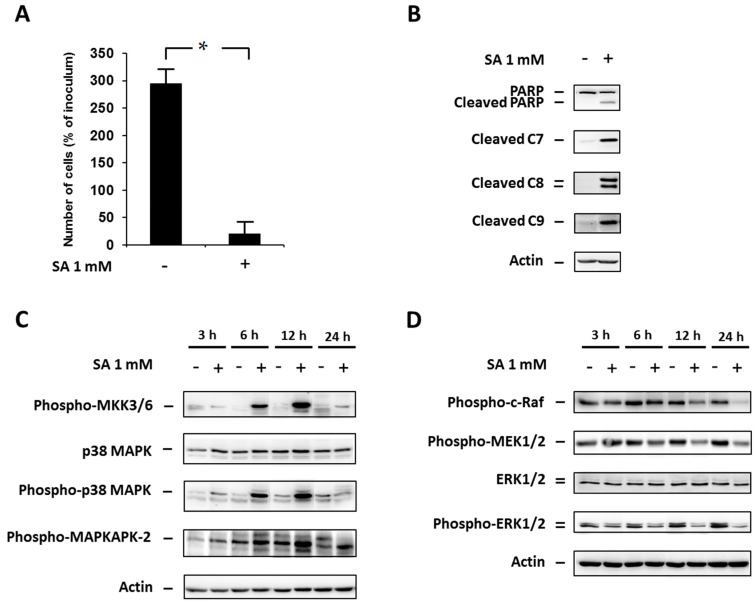
Effect of 1 mM stearic acid (SA) (see “Materials and Methods”) on (**A**) cell growth and viability; (**B**) the level of cleaved PARP, caspase-7 (C7), caspase-8 (C8) and caspase-9 (C9) (markers of apoptosis); (**C**) the level of phospho-MKK3/6, p38 MAPK, phospho-p38 MAPK, phospho-MAPKAPK-2 (p38 MAPK signaling pathway); and (**D**) the level of phospho-c-Raf, phospho-MEK1/2, ERK1/2, phospho-ERK1/2 (the ERK signaling pathway) in NES2Y cells. Cells incubated without SA represented control cells. After 18 h of incubation (see “Materials and Methods”) for markers of apoptosis (**B**) and 3, 6, 12 and 24 h of incubation for p38 MAPK and ERK pathways members (**C**,**D**), the levels of individual proteins were determined using Western blot analysis and the relevant antibodies (see “Materials and Methods”). Monoclonal antibody against human actin was used to confirm equal protein loading. The data shown were obtained in one representative experiment from at least three independent experiments. When assessing cell growth and viability (**A**), cells were seeded at a concentration of 2 × 10^4^ cells/100 µL of culture medium per well of 96-well plate (see “Materials and Methods”). The number of living cells was determined after 48 h of incubation. Each column represents the mean of four separate cultures ± standard error of the mean (SEM). * *p* < 0.05 when comparing the number of control cells and cells treated with SA.

We assessed cell viability as well as cleavage of PARP (a common marker of apoptosis) and caspase-7, -8 and -9 activation by cleavage after SA (1 mM) exposure, in NES2Y cells. SA application resulted in significant induction of cell death within 48 h after SA application (Figure 1A), and increased caspase-7, -8, -9 and PARP (a substrate of activated executioner caspases) cleavage 18 h after SA application (Figure 1B). Previously, it was found that there is nearly no activation of caspase-3 in NES2Y cells after SA exposure, and that caspase-2 does not play a key role in SA-induced apoptosis [8,29].

Next, we assessed the levels of activated (phosphorylated) members of the p38 MAPK signaling pathway (phospho-MKK3/6, phospho-p38 MAPK, phospho-MAPKAPK-2) as well as the levels of activated members of the ERK signaling pathway (phospho-c-Raf, phospho-MEK1/2, phospho-ERK1/2) within 24 h after SA application, in NES2Y cells.

SA treatment resulted in an increase in the level of phosphorylated members of the p38 MAPK pathway as early as 3 h after application. The level of phosphorylation increased to a maximum at 12 h after application for all tested proteins. At 24 h after treatment, the level of phosphorylation decreased. No change was detected in the level of total p38 MAPK during 24 h after SA application (Figure 1C).

Levels of phosphorylated members of the ERK pathway decreased as early as 3 h after SA application, except for MEK1/2. The effect of SA increased to the maximum for all tested proteins 12–24 h after application. We did not detect any change in the level of total ERK1/2 during 24 h after SA application (Figure 1D).

### 2.2. Effect of p38 MAPK Silencing

In order to test the involvement of p38 MAPK in apoptosis signaling induced by SA in NES2Y cells, we assessed the effect of p38 MAPK silencing by specific siRNA on cell growth and viability after SA treatment. We also tested the effect of p38 MAPK silencing on phosphorylation of MAPKAPK-2 (pathway member downstream of p38 MAPK) and phosphorylation of ERK pathway members (c-Raf, MEK1/2 and ERK1/2) after SA application. To assess the efficiency of silencing, we measured the level of total p38 MAPK and phospho-p38 MAPK, respectively.

**Figure 2:**
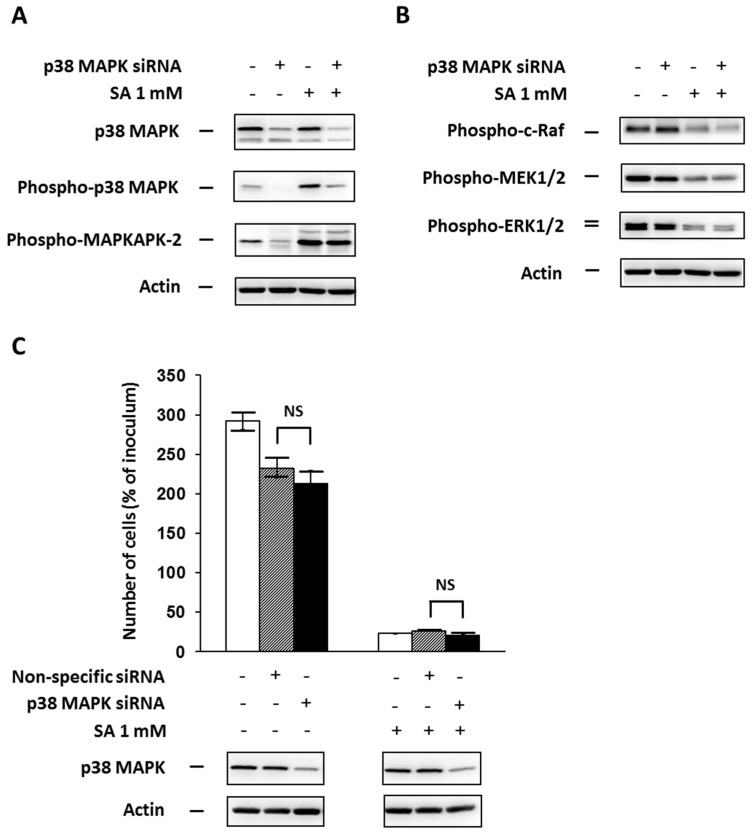
Effect of p38 MAPK silencing, using a specific siRNA (see “Materials and Methods”) and the effect of stearic acid (SA), on (**A**) the level of p38 MAPK, phospho-p38 MAPK, phospho-MAPKAPK-2 (substrate of p38 MAPK); (**B**) the level of phospho-c-Raf, phospho-MEK1/2, phospho-ERK1/2 (the ERK signaling pathway); and (**C**) cell growth and viability of NES2Y cells. Cells incubated without siRNA represented control cells. After 18 h of incubation (see “Materials and Methods”) with or without stearic acid (SA) (**A**,**B**), the level of individual proteins was determined using Western blot analysis and the relevant antibodies (see “Materials and Methods”). A monoclonal antibody against human actin was used to confirm equal protein loading. The data shown were obtained in one representative experiment from three independent experiments. When assessing cell growth and viability (**C**), cells were seeded at a concentration of 2 × 10^4^ cells/100 µL of culture medium per well of 96-well plate (see “Materials and Methods”). The number of living cells was determined after 48 h of incubation with or without SA. Non-specific siRNA was used as a negative control. Each column represents the mean of four separate cultures ± SEM. NS (non-significant) when comparing the number of cells incubated with p38 MAPK specific siRNA and with non-specific siRNA.

p38 MAPK silencing (approximately 60%) resulted in a decrease in phospho-p38 MAPK level, which was expected, and also a decrease in phospho-MAPKAPK-2 level 18 h after SA application (Figure 2A). However, it had nearly no effect on the level of phosphorylated ERK pathway members (Figure 2B). Cell viability was not significantly affected by p38 MAPK silencing during 48 h after SA treatment (Figure 2C)

### 2.3. Effect of the Specific p38 MAPK Inhibitor SB202190

We also assessed the effect of inhibition of p38 MAPK activity, using the specific inhibitor SB202190, on cell growth and viability, phosphorylation of MAPKAPK-2 (pathway member downstream of p38 MAPK), and phosphorylation of ERK pathway members (c-Raf, MEK1/2 and ERK1/2) after SA treatment in NES2Y β-cells. To assess the efficiency of inhibition, we measured the level of phospho-MAPKAPK-2.

**Figure 3:**
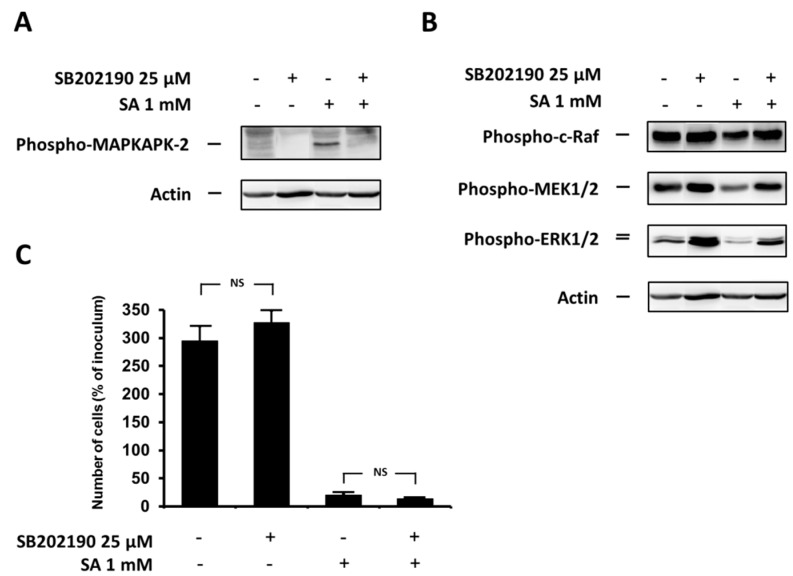
Effect of the specific p38 MAPK inhibitor, SB202190, (see “Materials and Methods”) and the effect of stearic acid (SA) on (**A**) the level of phospho-MAPKAPK-2 (substrate of p38 MAPK); (**B**) the level of phospho-c-Raf, phospho-MEK1/2, phospho-ERK1/2 (the ERK signaling pathway); and (**C**) cell growth and viability of NES2Y cells. Cells incubated without the inhibitor represented control cells. After 12 h of incubation (see “Materials and Methods”) (**A**,**B**), the level of individual proteins was determined using Western blot analysis and the relevant antibodies (see “Materials and Methods”). A monoclonal antibody against human actin was used to confirm equal protein loading. The data shown were obtained in one representative experiment from three independent experiments. When assessing cell growth and viability (**C**), cells were seeded at a concentration of 2 × 10^4^ cells/100 µL of culture medium per well of 96-well plate (see “Materials and Methods”). The number of living cells was determined after 48 h of incubation. Each column represents the mean of four separate cultures ± SEM. NS (non-significant) when comparing the number of control cells and cells treated with SB202190 as well as when comparing the effect of SA alone and applied together with SB202190.

p38 MAPK inhibition resulted in a decrease in phospho-MAPKAPK-2 level (Figure 3A) and in an increase of the levels of phosphorylated ERK pathway members (Figure 3B) 12 h after SA application. p38 MAPK inhibition in cells without SA exposure also increased the levels of phosphorylated ERK pathway members (Figure 3B). Cell viability was not significantly affected by the p38 MAPK inhibition within the 48-h period after SA treatment (Figure 3C).

### 2.4. Effect of p38 MAPK Overexpression

Next, we assessed the effect of p38 MAPK overexpression, through specific plasmid transfection, on cell growth and viability, phosphorylation of MAPKAPK-2 (pathway member downstream of p38 MAPK), and phosphorylation of ERK pathway members (c-Raf, MEK1/2 and ERK1/2) after SA exposure in NES2Y β-cells. To assess the efficiency of overexpression, we measured the level of total p38 MAPK and phospho-p38 MAPK, respectively.

**Figure 4:**
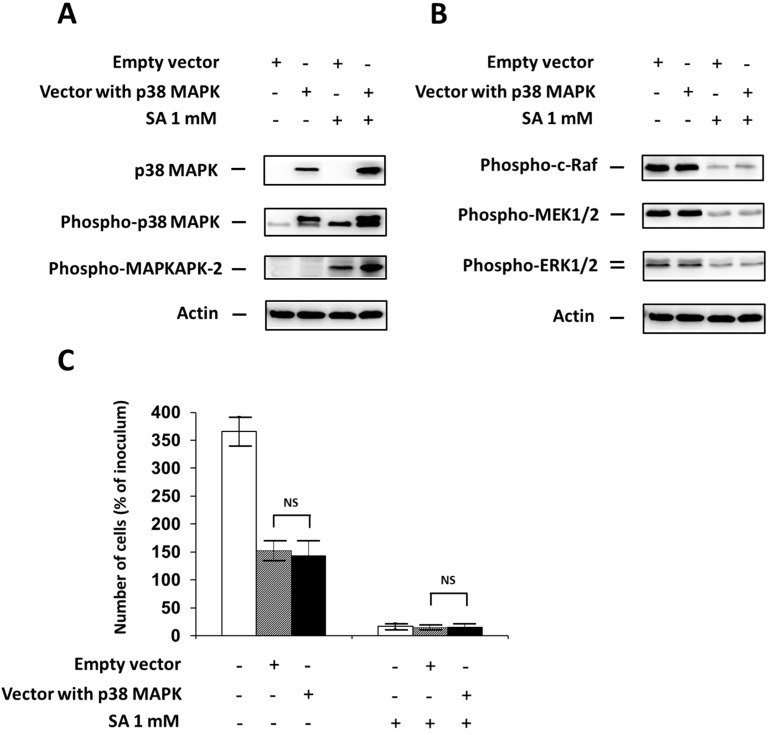
Effect of p38 MAPK overexpression, using transfection with a specific plasmid (Vector with p38 MAPK) (see “Materials and Methods”) and the effect of stearic acid (SA) on (**A**) the level of p38 MAPK, phospho-p38 MAPK and phospho-MAPKAPK-2 (substrate of p38 MAPK); (**B**) the level of phospho-c-Raf, phospho-MEK1/2, phospho-ERK1/2 (the ERK signaling pathway); and (**C**) cell growth and viability of NES2Y cells. Cells transfected with an empty vector (Empty vector) represented control cells. After 18 h of incubation (see “Materials and Methods”) with or without stearic acid (SA) (**A**,**B**), the level of individual proteins was determined using Western blot analysis and the relevant antibodies (see “Materials and Methods”). A monoclonal antibody against human actin was used to confirm equal protein loading. The data shown were obtained in one representative experiment from three independent experiments. The fact that the band of p38 MAPK in the control samples is not visible here resulted from a large difference in p38 MAPK content in control and transfected cells. When assessing cell growth and viability (**C**), cells were seeded at a concentration of 2 × 10^4^ cells/100 µL of culture medium per well of 96-well plate (see “Materials and Methods”). The number of living cells was determined after 48 h of incubation with or without SA. Each column represents the mean of four separate cultures ± SEM. NS (non-significant) when comparing the number of cells incubated with plasmid DNA containing p38 MAPK (Vector with p38 MAPK) and cells incubated with empty plasmid DNA (empty vector).

Significant p38 MAPK overexpression resulted in an expected increase in phospho-p38 MAPK level and also an increase in phospho-MAPKAPK-2 level 18 h after SA application (Figure 4A). However, it had no effect on the level of phosphorylated ERK pathway members (Figure 4B). Cell viability was not significantly affected by p38 MAPK overexpression during 48 h after SA treatment (Figure 4C).

### 2.5. Effect of the Specific p38 MAPK Activator Anisomycin

Lastly, we also assessed the effect of p38 MAPK activation, using the specific activator anisomycin, on cell growth and viability, phosphorylation of MAPKAPK-2 (pathway member downstream of p38 MAPK), and phosphorylation of ERK pathway members (c-Raf, MEK1/2 and ERK1/2) in NES2Y β-cells. To assess the efficiency of activation, we measured the level of phospho-p38 MAPK as well as the level of phospho-MAPKAPK-2.

**Figure 5:**
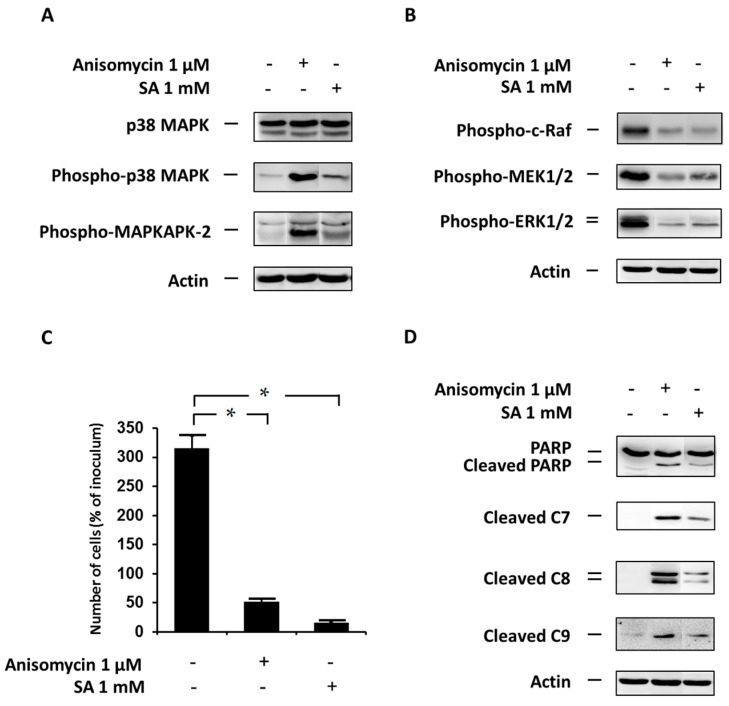
Effect of the specific p38 MAPK activator, anisomycin, (see “Materials and Methods”) and the effect of stearic acid (SA) on (**A**) the level of p38 MAPK, phospho-p38 MAPK and phospho-MAPKAPK-2 (substrate of p38 MAPK); (**B**) the level of phospho-c-Raf, phospho-MEK1/2, phospho-ERK1/2 (the ERK signaling pathway); (**C**) cell growth and viability; and (**D**) cleavage of PARP, caspase-7 (C7), caspase-8 (C8) and caspase-9 (C9) (markers of apoptosis) in NES2Y cells. Cells incubated without the activator and SA represented control cells. After 12 h of incubation (see “Materials and Methods”) (**A**,**B**,**D**), the level of individual proteins was determined using western blot analysis and the relevant antibodies (see “Materials and Methods”). A monoclonal antibody against human actin was used to confirm equal protein loading. The data shown were obtained in one representative experiment from three independent experiments. When assessing cell growth and viability (**C**), cells were seeded at a concentration of 2 × 10^4^ cells/100 µL of culture medium per well of 96-well plate (see “Materials and Methods”). The number of living cells was determined after 48 h of incubation. Each column represents the mean of four separate cultures ± SEM. * *p* < 0.05 when comparing the number of control cells and cells with anisomycin or SA.

Anisomycin-induced p38 MAPK activation resulted in a strong activation (phosphorylation) of MAPKAPK-2 (Figure 5A), 12 h after treatment. This activation appeared to be stronger than SA-induced activation. Anisomycin-induced p38 MAPK activation also led to a decrease in levels of the phosphorylated ERK pathway members, similar to that seen in SA-treated cells (Figure 5B). Furthermore, p38 MAPK activation resulted in induction of cell death (Figure 5C) within 48 h after anisomycin application, again similar to that seen in SA-treated cells.

Because cell death was induced, we also tested the effect of p38 MAPK activation on the cleavage of PARP (a common marker of apoptosis) and caspase-7, -8 and -9 activation. Caspase-7, -8, -9 activation as well as PARP cleavage was detected 12 h after activator application (Figure 5D).

## 3. Discussion

Our previous papers as well as this study demonstrate that saturated fatty acids (palmitic and stearic acid) induced apoptosis in the human pancreatic β-cell line NES2Y (see Figure 1A,B) [5,8,29]. Molecular mechanisms of apoptosis induction by saturated fatty acids (FAs) in β-cells have not been fully elucidated yet [9]. Some studies indicated a possible involvement of various kinases in pro-apoptotic signaling (e.g., [10,27,30]). In this study, we tested the involvement of the p38 MAPK signaling pathway in stearic acid (SA)-induced apoptosis in the human pancreatic β-cells NES2Y. Furthermore, this study showed that, together with p38 MAPK pathway activation, apoptosis-inducing SA also inhibits the ERK pathway in NES2Y cells. Thus, we also tested crosstalk between p38 MAPK pathway activation and ERK pathway inhibition after SA application. To our knowledge, there is no detailed study considering deeply the effect of saturated FAs on p38 MAPK signaling pathway in relation to apoptosis induction in β-cells of human origin. Moreover, the effect of stearate is studied only rarely despite it appears to be more effective than palmitate in human β-cells [8,31]. Human NES2Y cells respond to FAs similarly like primary human β-cells [2,32,33,34], as we also found in our previous experiments [5,8]. Thus, it may be expected that the responses to saturated SA seen in this study with NES2Y cells could be more or less relevant to its effects on human β-cells *in vivo*.

As mentioned above, we demonstrated that apoptosis-inducing SA activates the p38 MAPK pathway in NES2Y β-cells (see Figure 1C). Activation of p38 MAPK in response to palmitic acid has already been documented in NIT-1 and RIN pancreatic β-cells [10,18]. Some studies [35,36] as well as our preliminary data indicate that SA-induced pro-apoptotic signaling could begin on the plasma membrane of cells probably due to SA effects on membrane fluidity. It is likely that it happens upstream of p38 MAPK signaling. The effect of SA on membrane fluidity can result in changes in the activity of some membrane receptor(s) or membrane-associated protein(s), which can mediate further signaling.

We also demonstrated that apoptosis-inducing SA inhibits the ERK pathway in NES2Y β-cells (see Figure 1D). Inhibition of ERK1/2 activity, in response to palmitic acid, has already been documented in some studies with pancreatic β-cell lines [27,28]. However, in other papers, activation of ERK1/2 was shown [37,38]. Moreover, the experimental conditions used in these studies have been quite dissimilar. As with the effect of SA on p38 MAPK activation, the effect of SA probably starts upstream of the ERK pathway. Some papers [16,20] suggest that a possible regulator of this pathway might be p38 MAPK kinase. In this paper, we showed that p38 MAPK kinase was activated by SA (see above).

Inhibition of p38 MAPK expression by siRNA silencing and inhibition of p38 MAPK activity by the specific inhibitor SB202190 after SA application, as well as p38 MAPK overexpression using plasmid transfection, had no significant effect on cell viability (see Figure 2C, Figure 3C and Figure 4C). However, the application of the specific p38 MAPK activator, anisomycin, resulted in apoptosis induction similar to that seen after application of SA (see Figure 5C). It also resulted in PARP cleavage and caspase-7, -8, -9 activation (see Figure 5D).

The insignificant effect of p38 MAPK silencing on cell viability after SA treatment could have resulted from incomplete inhibition of p38 MAPK expression (see Figure 2A). The remaining amount of p38 MAPK was still able to transduce a sufficient signal for apoptosis induction. The second possibility of explanation is that the p38 MAPK signaling pathway does not play a key role in apoptosis induction after SA treatment. The insignificant effect of the inhibitor application on cell viability could be explained by a similar way like the insignificant effect of p38 MAPK silencing. The insignificant effect of p38 MAPK overexpression, accompanied by an increased level of phospho-p38 MAPK (see Figure 4A), rather supports the possibility that the p38 MAPK pathway does not play a key role.

Concerning the effect of the activator, it should be mentioned that anisomycin also has the potential to affect, besides p38 MAPK, the activation of other molecules. Thus, the described effect of the activator on cell viability may not be mediated by p38 MAPK. Nonetheless, there is a possibility that the p38 MAPK pathway is somehow involved in SA-induced pro-apoptotic signaling in NES2Y cells. However, it probably does not represent the main pathway of the pro-apoptotic SA effect. The possibility of a pro-apoptotic role for p38 MAPK has also been mentioned in other types of pancreatic β-cells or islets exposed to saturated FAs [10,18,39]. Nevertheless, in these cells, p38 MAPK seems to be more important as a mediator of saturated FA-induced apoptosis.

We can just speculate as to possible mechanisms playing a substantial role in saturated FA-induced apoptosis in pancreatic β-cells. These mechanisms probably represent a network of multiple signaling pathways. It is clear that caspases are involved here [8,32,40,41]. Generally, the involvement of the mitochondrial as well as the receptor pathways of apoptosis induction was documented in pancreatic β-cell lines or islets of animal or human origin (e.g., [2,40,42,43,44,45]). The involvement of inducible forms of nitric oxide synthase activation and nitric oxide production seems also speculative [1,6,46,47]. Experimental evidence strongly supports the role of *de novo* ceramide formation in saturated FA-induced apoptosis [2,42,46]. Other considered mechanisms that might play a role in regulation of β-cell viability by saturated FAs are activation of protein kinase Cδ [30], degradation of carboxypeptidase E [48], calpain-10 activation [41], activation of the transcription factor NF-κB [49,50], inhibition of protein kinase B [40], and the level of stearoyl-CoA desaturase-1 expression [33,51]. Nevertheless, the most studied molecular mechanism suggested to mediate FA-induced apoptosis is signaling of endoplasmic reticulum stress [7,29,32,34,49,52,53].

p38 MAPK silencing had no significant effect on ERK pathway activation (see Figure 2B). This could again be the result of incomplete inhibition of p38 MAPK expression (see above). On the other hand, application of the p38 MAPK inhibitor, SB202190, resulted in recognizable activation of ERK pathway members (see Figure 3B). However, it should be mentioned that this inhibitor effect could be the result of a direct effect of the inhibitor on the ERK pathway, since activation of c-Raf by SB202190 has been documented [54].

No significant effect on ERK pathway activation was also detected after p38 MAPK overexpression (see Figure 4B) while the application of p38 MAPK activator, anisomycin, resulted in significant inhibition of activation of ERK pathway members (see Figure 5B). Unfortunately, no significant effect of p38 MAPK overexpression, accompanied by increased level of phospho-p38 MAPK (see Figure 4A), on ERK pathway activation does not support the possibility of crosstalk. Regarding the effect of the activator, it should be noted that the effect of anisomycin on ERK pathway activation might not necessarily be mediated by p38 MAPK, since the activator can also affect other molecules. Although some of the approaches used to regulate p38 MAPK activation had no significant effect on ERK pathway activation; it seems that p38 MAPK kinase activation has an inhibitory effect on the ERK pathway in NES2Y β-cells after SA application. To date, no data documenting possible crosstalk between the p38 MAPK pathway and the ERK pathway, in pancreatic β-cells, has been published.

Taken together, we demonstrated that SA at apoptosis-inducing concentrations activates the p38 MAPK signaling pathway in human β-cells. We suggest that the activation of the p38 MAPK signaling pathway could be somehow involved in apoptosis induction by SA. However, this involvement does not seem to play a key role. Crosstalk between p38 MAPK pathway activation and the accompanying inhibition of the ERK signaling pathway after SA application seems more likely. Thus, the ERK pathway inhibition by p38 MAPK activation does not also seem to be essential for SA-induced apoptosis in human β-cells.

## 4. Materials and Methods

### 4.1. Materials

All chemicals were from Sigma-Aldrich (St. Louis, MO, USA), unless otherwise stated. For western blot analysis, the following primary and secondary antibodies were used: anti-phospho-MKK3/6 (#9236), anti-p38 MAPK (#8690), anti-phospho-p38 MAPK (#4511), anti-phospho-MAPKAPK-2 (#3007), anti-phospho-c-Raf (#9427), anti-phospho-MEK1/2 (#9154), anti-ERK1/2 (#5013), anti-phospho-ERK1/2 (#4370), anti-PARP (#9542), anti-cleaved caspase-7 (#9491), anti-cleaved caspase-8 (#9496), anti-cleaved caspase-9 (#9505) from Cell Signaling Technology (Danvers, MA, USA) and anti-actin (clone AC-40).

### 4.2. Cells and Culture Conditions

The human pancreatic β-cell line NES2Y [5,55] was used. NES2Y cells are proliferating insulin-secreting cells with a defect in glucose responsiveness. Cells were routinely maintained in an RPMI 1640 based culture medium [56]. In experiments, a defined serum-free medium [57] supplemented with 1 mM SA bound to 2% FA-free bovine serum albumin (BSA) was used [5]. Stock solutions containing SA bound to 10% BSA in a serum-free medium were prepared as described previously [5] and diluted to the required concentration of SA and BSA prior to experiments. SA/BSA molar ratios used in experiments were lower than the ratios known to exceed the binding capacity of BSA [58].

Our previous studies showed that SA, at a concentration of 1 mM, induced cell death in most NES2Y cells within 48 h of application. Apoptotic cells appeared within 24 h after SA application [5,8,29]. Therefore, all assessments were performed within 24 h after SA application, except for the assessment of cell growth and viability.

### 4.3. Assessment of the Effect of Stearic Acid on Cell Growth and Viability

Cells were seeded at a concentration of 2 × 10^4^ cells/100 μL of culture media into the wells of 96-well plate. After a 24-h pre-incubation period (allowing cells to attach) the culture medium was replaced with a serum-free medium containing 2% BSA with or without SA. The control medium contained 2% BSA only. After 48 h of incubation, the number of living cells was determined using a hemocytometer counting system, after staining with trypan blue.

### 4.4. Western Blot Analysis

Cells (approximately 1 × 10^6^ cells per sample) were seeded and after a 24-h pre-incubation period (allowing cells to attach), the culture medium was replaced with a serum-free medium containing 2% BSA with or without SA. The control medium contained 2% BSA only. After the required incubation period, cells were harvested and Western blot analysis was performed as described previously [8]. All primary antibodies were used in a 1:1000 dilution. The chemiluminescent signal was detected using a Carestream Gel Logic 4000 PRO Imaging System equipped with Carestream Molecular Imaging Software (Carestream Health, New Haven, CT, USA), which was used for image acquisition.

### 4.5. p38 MAPK Silencing by siRNA Transfection

To silence p38 MAPK expression, p38 MAPK specific siRNA (s3586, Life Technologies, Carlsbad, CA, USA) and INTERFERin (PolyPlus-Transfection, Illkirch, France), as transfection reagent, were used according to the manufacturer’s instructions.

To silence p38 MAPK, 2.1 × 10^5^ cells/6 mL were seeded into Petri dishes (Ø 6 cm). After 24 h (allowing cells to attach), the culture medium was replaced with new culture medium with or without the p38 MAPK specific siRNA and transfection reagent. The siRNA and transfection reagent were diluted in Opti-MEM^®^ Reduced Serum Medium (Life Technologies) to a final concentration of 150 nM and 0.4%, respectively, prior to transfection. After 72 h of incubation, cells were harvested and seeded into six-well plates at a density of 1 × 10^6^ cells/2.5 mL per well. After 24 h (allowing cells to attach), the culture medium was replaced with a serum-free medium containing: (1) fresh siRNA, transfection reagent (at the same concentration used for the initial inhibition of p38 MAPK expression) and 2% BSA with or without SA; or (2) 2% BSA with or without SA (control media). After 18 h of treatment, cells were harvested and lysates were prepared for western blot analysis as described previously [8]. The efficiency of p38 MAPK expression silencing was tested in each experiment at the protein level using Western blot analysis.

### 4.6. Assessment of the Effect of p38 MAPK Silencing on Cell Growth and Viability

The experiment was set up in the same way as described in “p38 MAPK silencing by siRNA transfection” with the following modifications. After 72 h of incubation with or without the specific or non-specific siRNA and transfection reagent, cells were seeded at a concentration of 2 × 10^4^ cells/100 μL of relevant medium (see above) into the wells of 96-well plate. After 48 h of incubation with or without SA, the number of living cells was determined using a hemocytometer counting system after staining with trypan blue.

### 4.7. p38 MAPK Overexpression by Plasmid Transfection

In order to increase expression of p38 MAPK in NES2Y cells, transfection with plasmids containing p38 MAPK was performed. The plasmid was originally produced by Roger Davis (Howard Hughes Medical Institute, Chevy Chase, MD, USA). Subsequently, it was subcloned into pcDNA 3.1 by Jarmila Králová (Institute of Molecular Genetics of the ASCR, Prague, Czech Republic) and kindly donated to us (with permission from Davis).

Cells were seeded into 6-well plates at a density of 1 × 10^6^ cells/2.5 mL per well. After 24 h (allowing cells to attach), the culture medium was replaced with new culture medium containing 2.5 µg of plasmid DNA (empty plasmid or plasmid containing p38 MAPK) and Lipofectamine 3000 (Invitrogen, Paisley, UK) as a transfection reagent according to the manufacturer’s instructions. After 48 h, the culture medium was replaced with a serum-free medium containing 2% BSA with or without SA. After 18 h of the treatment, cells were harvested and lysates were prepared for Western blot analysis as described previously [8]. The efficiency of transfection was tested by analyzing the level of p38 MAPK using Western blot.

### 4.8. Assessment of the Effect of p38 MAPK Overexpression on Cell Growth and Viability

The experiment was set up in the same way as that described in “p38 MAPK overexpression by plasmid transfection” with the following modifications. Cells were seeded at a concentration of 2 × 10^4^ cells/100 μL of relevant medium (see above) into the wells of the 96-well plate. The amount of plasmid DNA used was 100 ng per well. After 48 h of incubation with or without SA, the number of living cells was determined using a hemocytometer counting system after staining with trypan blue.

### 4.9. Inhibitor and Activator Application

Cells (approximately 5 × 10^5^ cells per sample) were seeded and after a 24-h pre-incubation period (allowing cells to attach) the culture medium was replaced with: (1) a serum-free medium with or without the p38 MAPK inhibitor SB202190 (Abcam, Cambridge, UK) at a desired concentration; (2) a serum-free medium containing 2% BSA with or without the p38 MAPK activator anisomycin (Sigma Aldrich, St. Louis, MO, USA) at required concentration; or (3) a serum-free medium containing 2% BSA and SA. The control medium contained only 2% BSA and the *vehiculum* dimethyl sulfoxide (DMSO). After 1 h of inhibitor pre-treatment, 2% BSA with or without SA was added to achieve the required concentrations. After 12 h of incubation, the cells were harvested and lysates were prepared for Western blot analysis as described previously [8]. The concentration of inhibitor/activator, which was necessary for efficient p38 MAPK inhibition/activation, was determined by testing the effect of several inhibitor/activator concentrations on the level of phosphorylated p38 MAPK and/or MAPKAPK-2 (substrate of p38 MAPK). The duration of treatment with the inhibitor/activator was selected based on the time course of activation/inhibition of p38 MAPK after SA and SB202190/anisomycin application.

### 4.10. Assessment of the Effect of Inhibitor or Activator on Cell Growth and Viability

Cells were seeded at 2 × 10^4^ cells/100 μL of culture media (see above) into the wells of 96-well plate. The p38 MAPK inhibitor SB202190, the activator anisomycin, and SA were applied in the same way as described above (“Inhibitor and activator application”). After 48 h of incubation, the number of living cells was determined using a hemocytometer counting system after staining with trypan blue.

### 4.11. Statistical Analysis

The statistical significance of observed differences was determined using the Student´s *t*-test. *p* < 0.05 was considered statistically significant.
